# miRNA changes associated with differentiation of human embryonic stem cells into human retinal ganglion cells

**DOI:** 10.1038/s41598-024-83381-9

**Published:** 2024-12-30

**Authors:** Maryam Esmaeili, Daniel A. Smith, Ben Mead

**Affiliations:** 1 School of Optometry and Vision Sciences, Cardiff University, Cardiff, CF24 4HQ UK; 2 Wales Kidney Research Unit, School of Medicine, Cardiff University, Cardiff, CF14 4XN UK; 3 School of Medicine, Systems Immunity University Research Institute, Cardiff University, Cardiff, CF14 4XN UK

**Keywords:** Gene expression, Stem cells in the nervous system, Visual system, Neuroscience, Stem cells

## Abstract

miRNA, short non-coding RNA, are rapidly emerging as important regulators in cell homeostasis, as well as potential players in cellular degeneration. The latter has led to interest in them as both biomarkers and as potential therapeutics. Retinal ganglion cells (RGC), whose axons connect the eye to the brain, are central nervous system cells of great interest, yet their study is largely restricted to animals due to the difficulty in obtaining healthy human RGC. Using a CRISPR/Cas9-based reporter embryonic stem cell line, human RGC were generated and their miRNA profile characterized using NanoString miRNA assays. We identified a variety of retinal specific miRNA upregulated in ESC-derived RGC, with half of the most abundant miRNA also detectable in purified rat RGC. Several miRNA were however identified to be unique to RGC from human. The findings show which miRNA are abundant in RGC and the limited congruence with animal derived RGC. These data could be used to understand miRNA’s role in RGC function, as well as potential biomarkers or therapies in retinal diseases involving RGC degeneration.

## Introduction

Retinal ganglion cells (RGCs) are the projection neurons of the retina, connecting the eye to the rest of the brain. RGCs represent a significant research interest for several reasons. Firstly, vision is a complex sense, with processing beginning in the retina and is itself a subject of intense scientific research. Secondly, a leading cause of irreversible blindness is the loss of RGCs and includes traumatic optic neuropathy and glaucoma. RGC research allows a deeper understanding of the mechanisms of RGC death as well as the potential neuroprotective effects of candidate treatments. Finally, RGCs are also arguably the most easily accessible central nervous system (CNS) neurons and understanding the intrinsic mechanisms behind axon regenerative failure could not only benefit victims of sight loss but also provide therapeutic strategies for those paralysed from spinal cord injury.

While there is a myriad of in vitro and in vivo techniques to study RGCs^1^, these are strictly animal-based as the study of human RGCs presents significant challenges. The tissue is inaccessible and so studies must rely on human RGCs from cadavers, that will have gone through 6–24 h of degeneration before it reaches the hands of researchers^2^. Given how rapid the neurodegenerative process is, this creates an unknowable “black box”, whereby we know human RGCs degenerate, but lack the means to understand the intricate processes responsible. To address this, researchers have begun using stem cell technology to generate human RGC in a dish. Embryonic and induced-pluripotent stem cells (ESC/iPSC) can be reliably differentiated into RGC, and subsequently studied. Perhaps one of the most well studied of these in recent years is the ESC-line generated in Donald Zack’s lab^3,4^, which have been CRISPR/Cas9 modified to express a fluorescent reporter gene, as well as a surface tag (Thy1), both under the control of an RGC-specific promotor (BRN3B). These cells have been used in a variety of ways including the testing of therapeutic compounds^5^, understanding the chemotropic cues associated with CNS/RGC axon regeneration^6^, or as donors to test the feasibility of RGC transplantation^7^.

The following study sought to determine the miRNA expression of human ESCs and the changes associated with differentiation into RGC. We also examined the miRNA changes of these two cell types while in culture for 48 h, to determine the reliability of their expression as phenotypic. The role of miRNA is complex, as unlike mRNA, in which the function of the translated protein can be investigated, each miRNA works by silencing 100 s of mRNA. Their ability to modulate and lower gene expression has led to suggestions that they act to buffer the gene expression of cells during moments of stress or environmental changes^8,9^. Their function is unequivocally necessary however, and silencing of key miRNA biogenesis factors is lethal^10^. The role of miRNA in RGC health and disease has been reviewed previously^11^, and animal studies have demonstrated that the miRNA profile of RGC differ substantially in different models of retinal disease^12^. Here we demonstrate the substantial miRNA changes associated with human RGC differentiation.

## Methods

### ESC culture and differentiation to RGC

All reagents were purchased from ThermoFisher unless otherwise specified.

ESC line used in this study was H7/H9 ESCs that had been CRISPR/Cas9 modified to express a fluorescent reporter gene (TdTomato), as well as a surface tag (Thy1.2), both under the control of an RGC-specific promotor (BRN3B), and were generously provided by Prof. Donald Zack^3,4^. ESC were expanded on Matrigel (#354230; Corning, NY, USA) with mTeSR1 Plus medium for 1–2 weeks (#100–0276; StemCell Technologies, Vancouver, Canada) and differentiated into RGC, as it has been previously described^1,3,4,13^. Briefly, five small molecules including Forskolin (#72114; StemCell Technologies), Dorsomorphin (#72102; StemCell Technologies), Inducer of definitive endoderm 2 (IDE2) (#72524; StemCell Technologies), nicotinamide (#N3376; Sigma, Allentown, PA, USA), and DAPT (D5942; Sigma) were added to hECS cultures, over a 40-day period, to generate RGC.

### RGC purification and preparation of cell lysates

Generated RGCs were purified from hESCs using mouse CD90.2 (Thy1.2) MicroBeads as per the manufacturer’s instructions (#130-121-278; Miltenyi Biotec, Auburn, CA, USA). Briefly, cells were detached using accutase solution (# A6964; Sigma) and the cell pellet resuspended in 390 µl of 0.5% BSA solution. RGCs were magnetically labelled with addition of 50 µl CD90.2 MicroBeads, incubated for 15 min at RT and mixed gently every 5 min. A 5 ml solution of 0.5% BSA was added to the mixture, followed by centrifuging at 130 g for 5 min. The cell pellet was resuspended in 400 µl 0.5% BSA by gentle pipetting. Positive selection of CD90.2^+^ was performed by magnetic separation using MS columns (#130-042-201; Miltenyi Biotec) in the magnetic field of OctoMACS™ separator (#130-042-109; Miltenyi Biotec). Having rinsed with 0.5% BSA, cell suspension was added to the columns, followed by three washes with 0.5% BSA. The magnetic labelled RGC was collected using a plunger and addition of lysis buffer and then vortex at full speed for 30 s.

### RNA isolation and nanostring nCounter microRNA expression analysis

Total RNA was isolated using the Qiagen® miRNeasy® advanced micro kit (# 217684; Qiagen, Hilden, Germany) as per the manufacturer’s instructions. The quality of RNA was assessed using an Agilent 2100 Bioanalyser (Agilent Technologies, Palo Alto, CA, USA) and all samples had an RNA Integrity Number (RIN) value of 9.70–10. 100 ng of input total RNA was profiled on a NanoString nCounter MAX platform using the Human v3 miRNA CodeSet kit (#150629; NanoSring, Seattle, WA, USA) to directly measure miRNA expression levels. Briefly, unique oligonucleotide tags were annealed and then ligated with the miRNAs of interest through a target specific bridge oligo followed by hybridization of the specific target of interest to the Reporter CodeSet. For this, samples were hybridized on a Veriti™ Thermal Cycler (Applied Biosystems, USA) for 20 h before being processed using the nCounter Prep Station followed by the nCounter Digital Analyser. Data normalization was performed using nSolver Analysis Software v4.0, whereby data was normalized to housekeeping genes (B2M, GAPDH, RPL19, RPLP0 and ACTB). Since the assay has a minimum detection threshold for weakly expressed miRNA, it was necessary to filter the normalized abundance values. As such, when comparisons between two groups are made, miRNA with abundance values of below 25 (in both groups) were removed. This applies to both the abundance measurements as well as the subsequent fold change heatmaps (whereby fold changes are presented as log2 fold change). Heatmaps further filtered out any average fold change that was not > 2 or < − 2.

### RT-qPCR

Confirmatory RT-qPCR was performed using TaqMan™ RT-qPCR reagents comparing ESC to RGC at 6 h (n = 3). In brief, RT for miRNA analysis in each sample extract was undertaken using the TaqMan™ high-capacity cDNA reverse transcription kit (4368813). Each reaction was prepared as a master mix containing 1.5 μL 10 × RT mix, 0.1 μL RNase inhibitor, 0.5 μL 25 × dNTP mix (100 mM), 3 μL miRNA-specific RT primer, 1 μL Multiscribe™ reverse transcriptase and 4.25 μL molecular biology grade water in each sample. 10 μL of this master mix was added to 0.1 mL microcentrifuge strips, followed by 5 μL of miRNA extract. Following mixing and collection by centrifugation, thermocycling was carried out at 4 °C for 5 min, 16 °C for 30 min and 42 °C for 30 min, followed by 85 °C for 5 min and finally maintained at 4 °C. Each cDNA sample was then diluted with 30 μL water to a final volume of 45 μL. Each qCPR reaction contained 10 μL TaqMan™ master mix II (no UNG), 1 μL TaqMan™ specific miRNA PCR primer, 5 μL water and 4 μL diluted cDNA. Finally these samples were analysed in 96 well fast plates (4346907) using a Quantstudio 7 Flex qPCR System.

TaqMan™ specific RT and qPCR primers for hsa-miR-873-3p and hsa-miR-93-5p were selected as references with stability values of 0.108 and 0.163, respectively, following analysis using Normfinder^14^. Having compared the Ct value of miRs to the references, data was normalised to hsa-miR-93-5p as the least variable sequence and the data reported as relative expression using the 2^^-ΔΔCt^ method.

## Results

### miRNA changes in ESC in culture

**Fig. 1:**
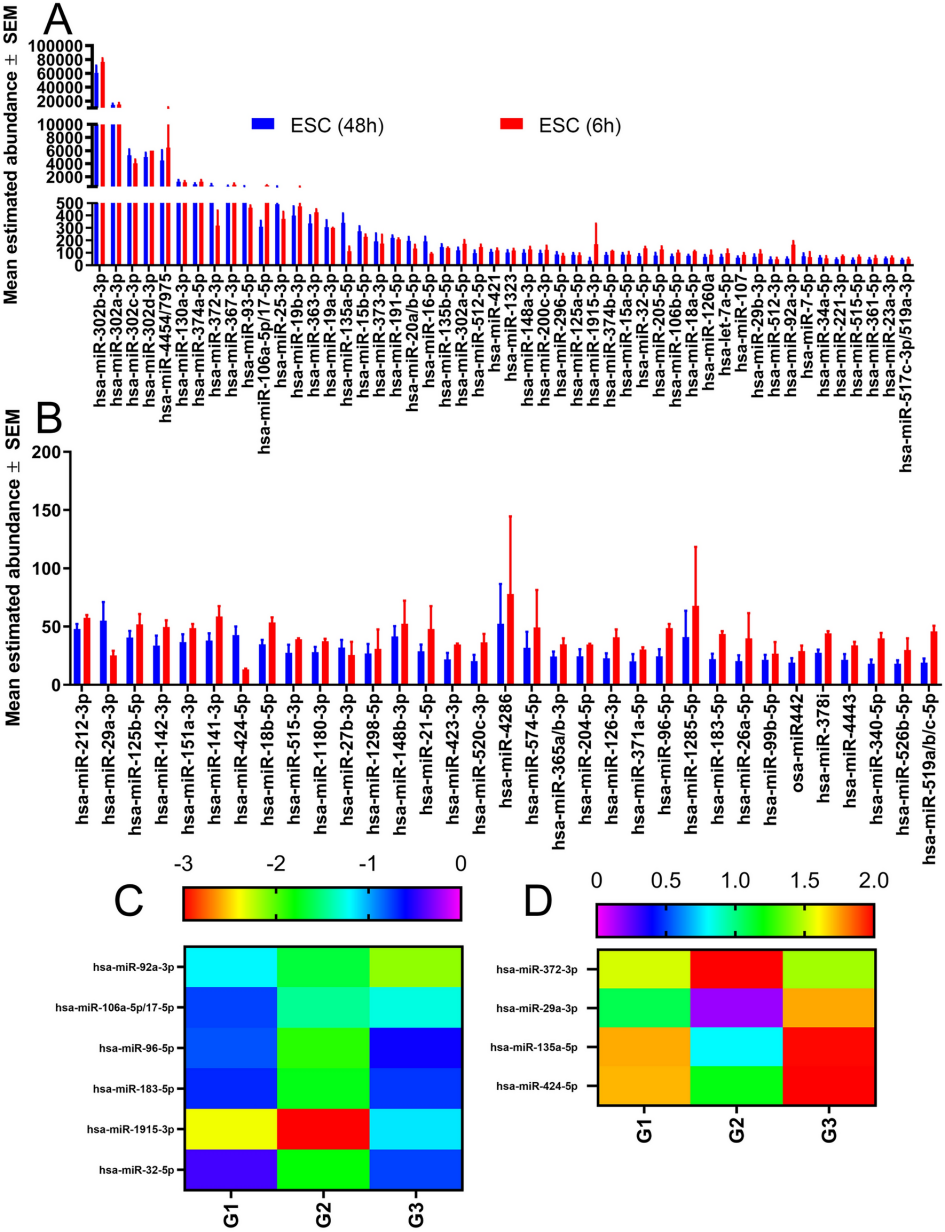
miRNA abundance and fold change heat map profiles of human embryonic stem cells (ESCs) in culture. (**A**,**B**) Histograms display the average (mean ± SEM; n = 3) abundance of miRNA in H7/H9 ESCs as determined by Nano String miRNA assay, sorted in descending order (note Y axis change in panel B). RNA was isolated from ESCs cultured for 48 (blue) and 6 (red) hours to determine miRNA drift. RNA abundances below 25 are excluded due to sensitivity limits of the assay. (**C**,**D**) Heatmaps display the log_2_ fold change of miRNA from ESC 48 h in culture compared to those 6 h in culture (n = 3). miRNA were selected from panel A/B that have an average <  −2 (C) or > 2 (D) fold change. G1-3 refers to Group 1 to 3.

While in culture, the CRISPR/Cas9 H7/H9 ESC remained relatively stable with few miRNA changes. A total of 83 miRNA were found to be expressed in ESC (above the noise threshold), 10 of which demonstrated at least a twofold change between two separate cultures (Fig. 1). Of these 10, miR-106a, -92a, and -424 were found to be statistically significant.

### miRNA changes in RGC following differentiation from ESC

**Fig. 2:**
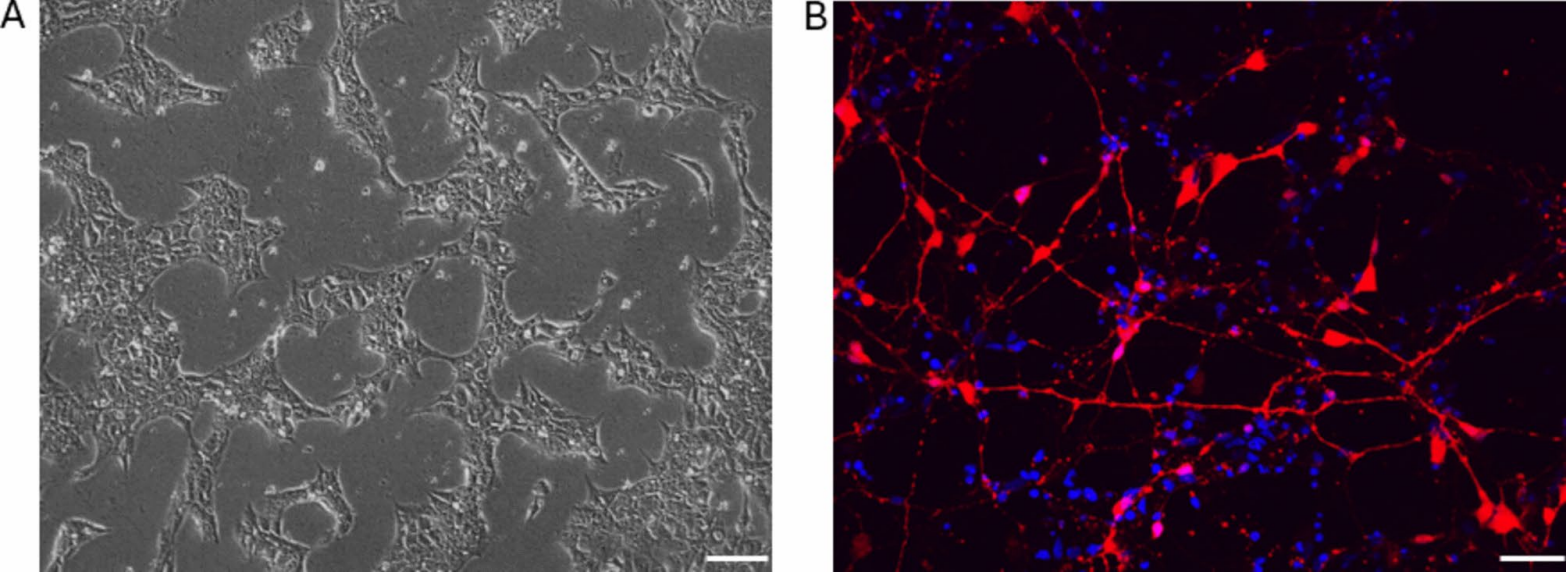
ESC culture and differentiation images. (**A**) ESC plated in matrigel coated plate with a density of 5 × 10^5, the density for when differentiation began, imaged using brightfield microscopy (scale bar: 100 µm). (**B**) ESC-induced RGC fixed with 4% PFA, counterstained with DAPI, and imaged using confocal microscopy, showing BRN3B-tdTomato (RGC phenotypic marker; red; scale bar: 50 µm).

**Fig. 3:**
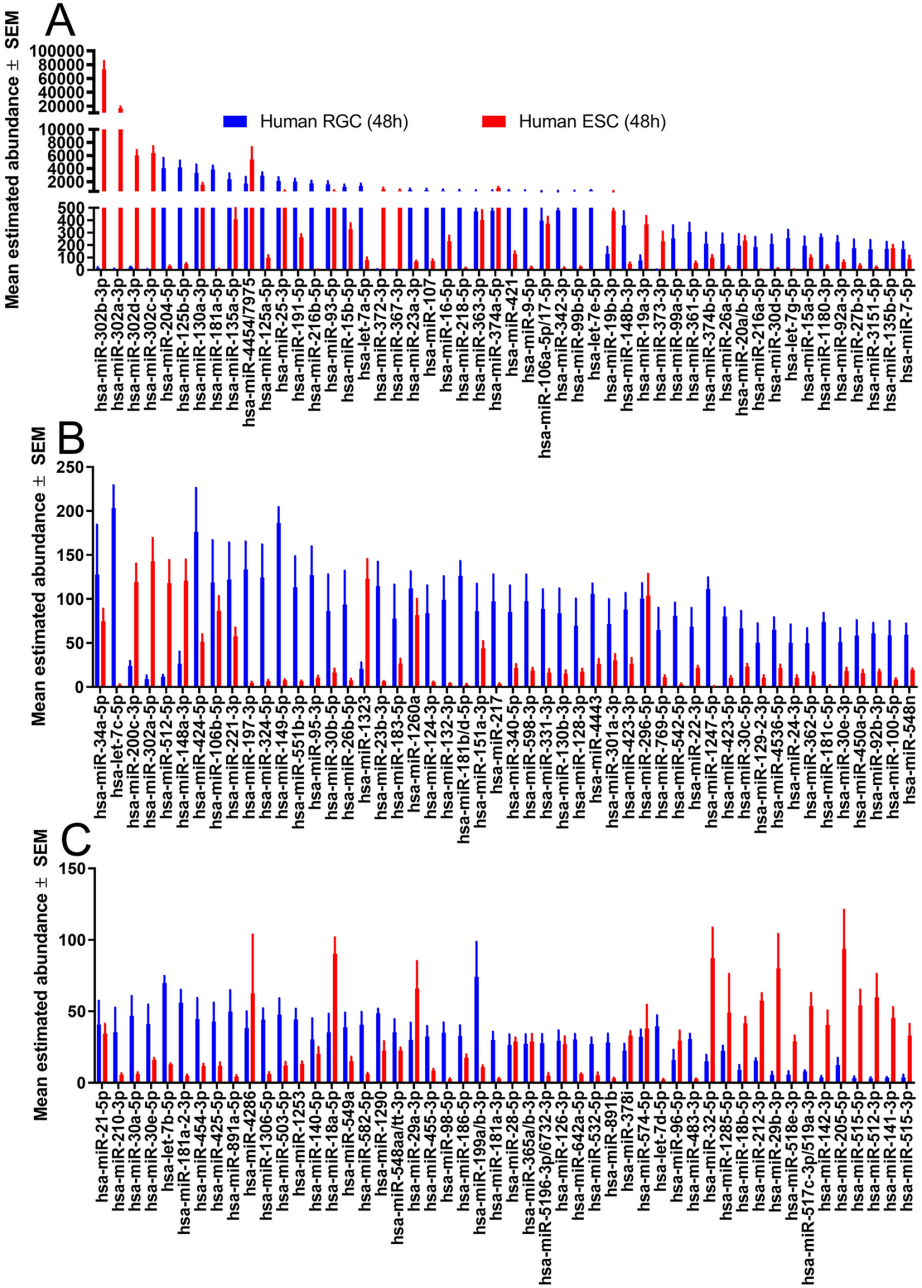
miRNA abundance of human embryonic stem cells (ESC) before and after differentiation into retinal ganglion cells (RGCs). **(A**–**C**) Histograms display the average (mean ± SEM; n = 3) abundance of miRNA in H7/H9 ESCs before and after successful differentiation into RGCs as determined by Nano String miRNA assay, sorted in descending order (note Y axis change in panel B and C). RNA was isolated from ESCs (red) and RGCs differentiated from the same ESCs (blue) to determine miRNA changes. RNA abundances below 25 are excluded due to sensitivity limits of the assay.

**Fig. 4:**
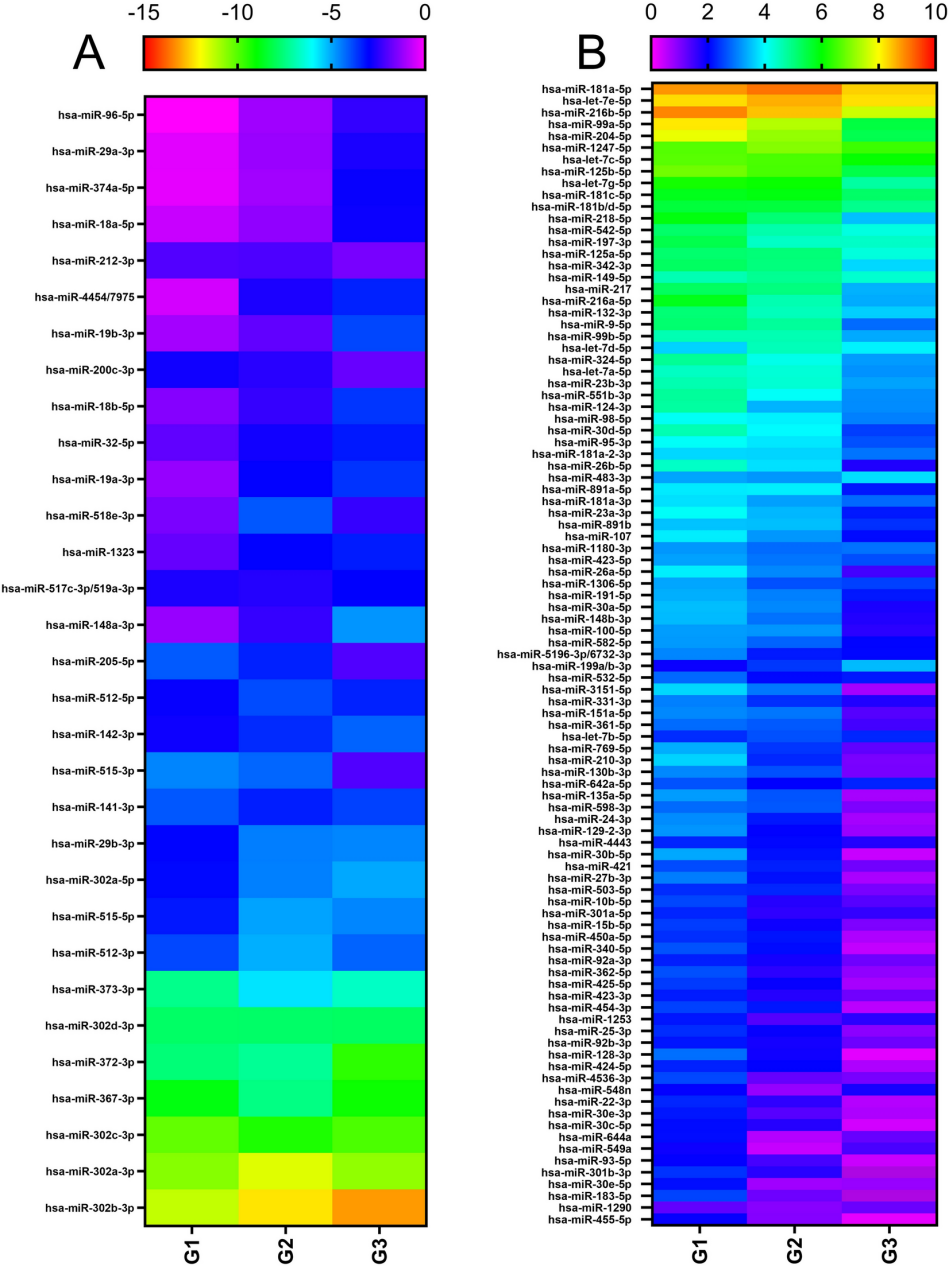
miRNA fold change heat map profiles of human embryonic stem cells (ESC) before and after differentiation into retinal ganglion cells (RGCs). (**A**,**B**) Heatmaps display the log_2_ fold change of miRNA from human RGC compared to human ESCs they were differentiated from (n = 3). miRNA were selected from Fig. 3 that have an average <  −2 (A) or > 2 (B) fold change. G1-3 refers to Group 1 to 3.

**Fig. 5:**
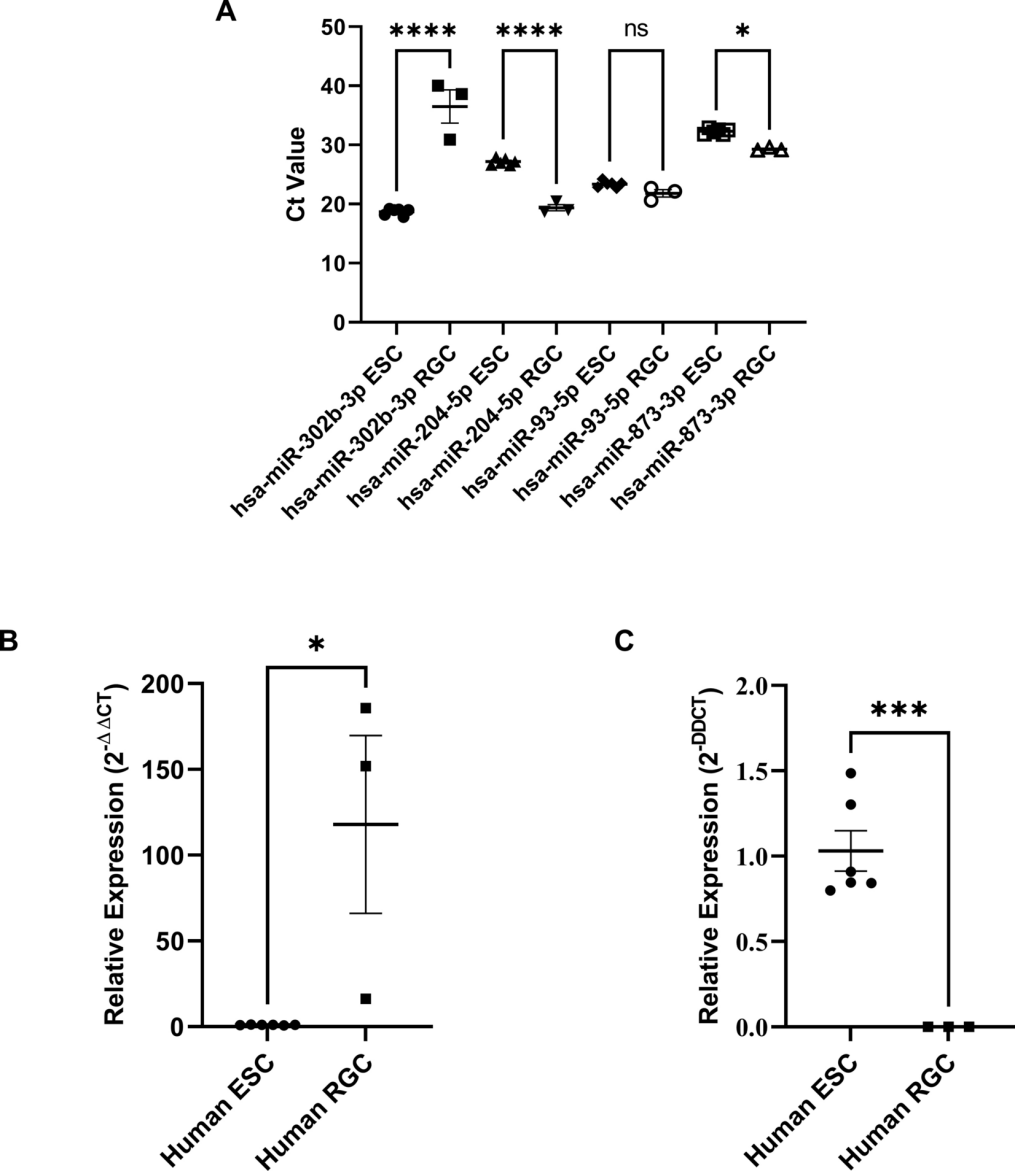
Confirmatory RT-qPCR analysis of 2 miRNAs of differing expression changes (**A**) Ct values of all 4 miRs analysed, 2 normalisers miR-93-5p and miR-873-3p, and 2 comparators miR-204-5p and miR-302b-3p. (**B**) Relative expression of miR-204-5p in ESC vs. RGC normalized to miR-93-5p. (**C**) Relative expression of miR-302b-3p in ESC vs. RGC normalized to miR-93-5p.

After differentiation of ESC into RGC (Fig. 2), RGC underwent significant changes in their miRNA expression. A total of 150 miRNA were found to be expressed in either ESC-derived RGC or ESC (above the noise threshold; Fig. 3), 128 of which demonstrated at least a twofold change between the two (Fig. 4). Of these 128, over half were found to be statistically significant. The nCounter expression trends were confirmed through RT-qPCR analysis of miR-204-5p and miR-302b-3p as an example of up and down-regulated miRNAs (Fig. 5).

### Predicted functional consequences associated with miRNA changes observed in RGC following differentiation from ESC as determined by in silico analysis

**Fig. 6:**
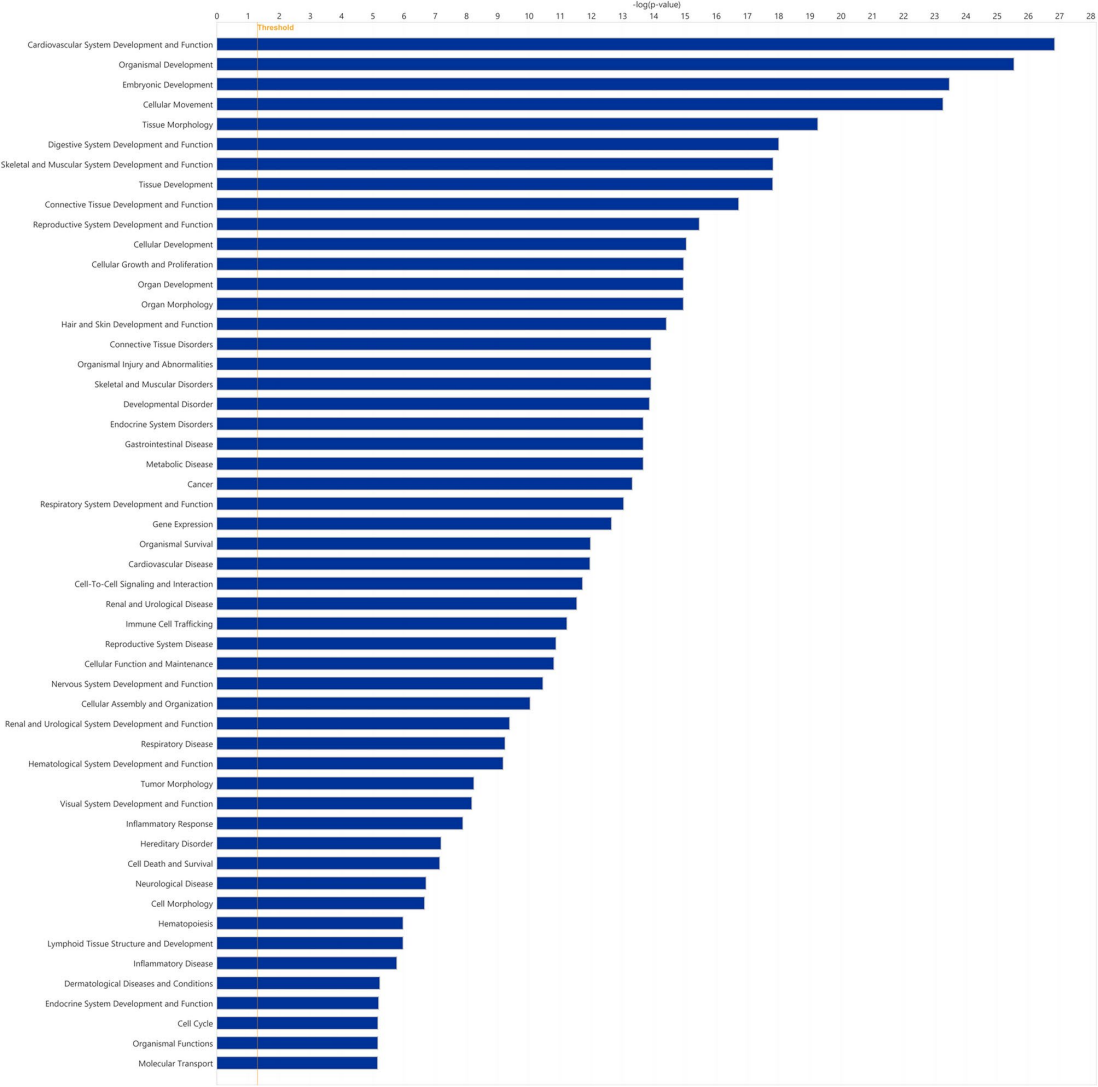
Ingenuity pathway analysis of all miRNAs identified in this study, relating these miRNA to their likely function.

Pathway analysis was performed using Ingeuintiy Pathway Analysis (Qiagen, Hilden, Germany). IPA analysis for identified miRNA presented in Fig. 3, highlighted the involvement of the miRNAs in mechanisms of organismal injury and abnormalities (112 miR) and neurological disorders (74 miR) along with cellular movement (58 miR), development (76 miR), growth and proliferation (70 miR) among others (Fig. 6). miR-204-5p and miR-302b-3p were abundantly upregulated in RGC and ESC, respectively. miR-204 plays a significant role in the regulation of RGC. This miRNA is involved in various cellular processes, including apoptosis^15^, oxidative stress response^16^, and neuroprotection^17^. These roles make miR-204 a potential target for therapeutic strategies aimed at treating retinal diseases and preserving vision. Moreover, miR-302 is known for its role in promoting cell proliferation^18^, and crucial for maintaining pluripotency in stem cells and affecting differentiation of progenitor cells into various cell types, including neurons^19^. The observed upregulation of miR-302 in our study is in line with this previous study. miR-302 enhances BMP signaling by targeting DAZAP2, SLAIN1, and TOB2 and blocking these genes can prevent the neural differentiation of human ESC^20^. Thus, miR-302 can be a suitable therapeutic agent for regenerating RGC in conditions such as optic neuropathies or glaucoma^21^.

### miRNA changes in ESC-derived RGC

**Fig. 7:**
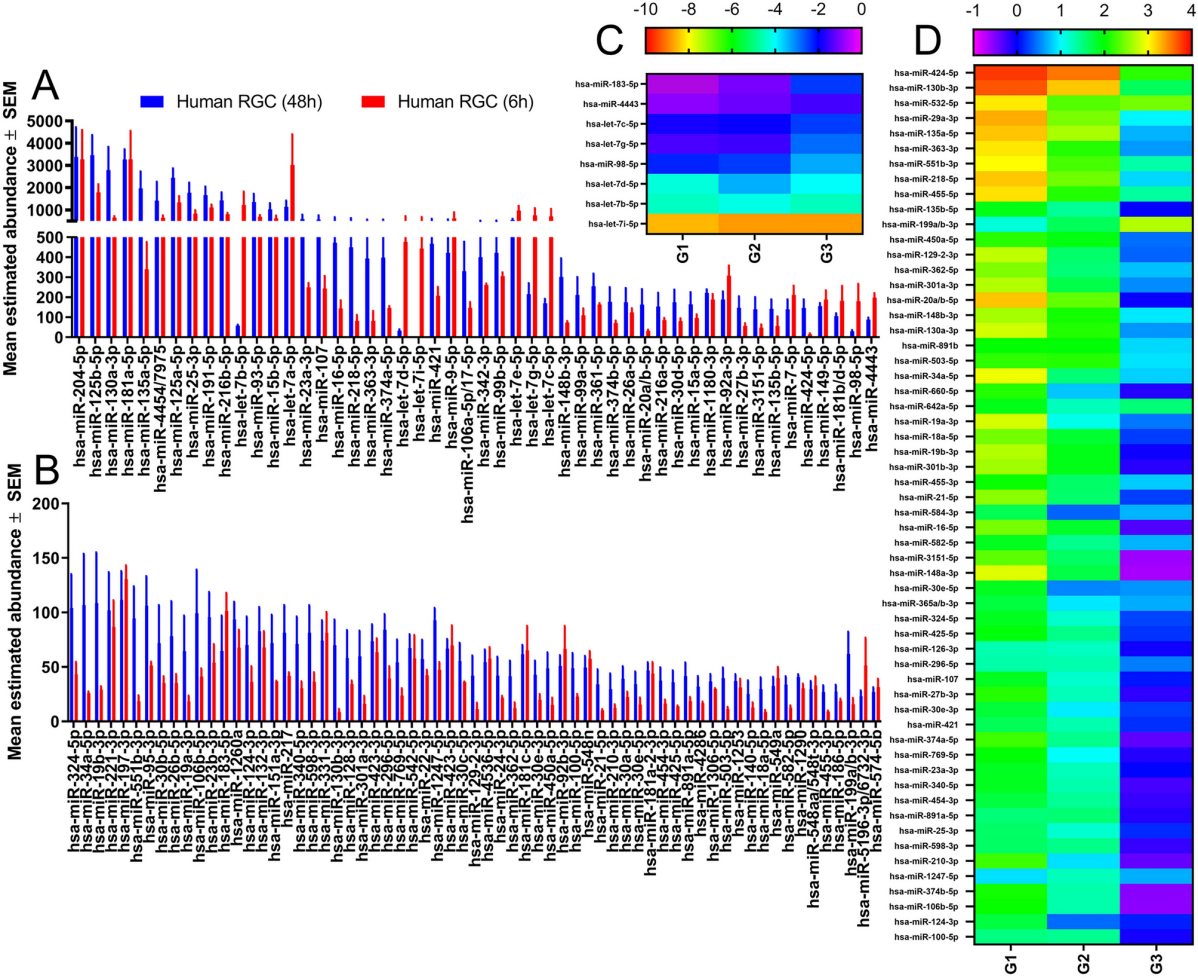
miRNA abundance and fold change heat map profiles of human retinal ganglion cells (RGCs) in culture. (**A**,**B**) Histograms display the average (mean ± SEM; n = 3) abundance of miRNA in ESC-derived RGCs as determined by Nano String miRNA assay, sorted in descending order (note Y axis change in panel B). RNA was isolated from RGCs cultured for 48 (blue) and 6 (red) hours to determine miRNA drift. RNA abundances below 25 are excluded due to sensitivity limits of the assay. (**C**,**D**) Heatmaps display the log_2_ fold change of miRNA from RGCs 48 h in culture compared to those 6 h in culture (n = 3). miRNA were selected from panel A/B that have an average <  −2 (C) or > 2 (D) fold change. G1-3 refers to Group 1 to 3.

While in culture, ESC-derived RGC were still undergoing significant changes with respect to their miRNA, A total of 115 miRNA were found to be expressed in ESC-derived RGC (above the noise threshold), 66 of which demonstrated at least a twofold change between 6 and 48 h in culture (Fig. 7). Of these 66, miR-424, -551, -130b, -4443, -1247, -503, -582, -29a, -455, -642a, -532 and -455 were found to be statistically significant.

## Discussion

The present study identified the miRNA expressed in human H7/H9 ESC, as well as their changes when they are differentiated into RGC using well established techniques. We looked at both abundance and log_2_ fold changes to understand the miRNA profile of these cells.

It has become apparent that miRNA play a significant role in both the healthy functioning of a cell (including RGC) as well as mediating the degenerative processes of an unhealthy one^11^. There is also evidence that miRNA can be exploited as powerful therapeutic strategies for degenerating RGC. Rather than having acute and substantial effects on cell biology, evidence suggests that miRNA are instead a buffering system, modulating gene expression to protect against large changes often caused by environmental and stressful challenges^8,9^. By understanding the miRNA profile of a cell type, a greater understanding of the role the miRNA play can be explored. We have previously profiled the miRNA of rodent RGC^12^, and thus build on this literature with a profile of human RGC, generated from ESC.

Our ESC remained relatively stable in culture, with only a small number of miRNA changing, and only by a small amount, with three showing statistical significance. All three of these, miR-106a^22,23^, miR-92a^24^, and miR-424^25^ were downregulated over time and have been linked to cell proliferation and migration and likely reflect the changing culture conditions as more cells occupy the flask. The miR-302 family of miRNA were the most abundant miRNA found in the ESC, and their role as a specific marker of ESC has already been established^26^, yet served as useful positive control of both the stemness of our cells, and its reduction, a marker of successful differentiation.

After differentiation, expectedly, there were substantial changes in the miRNA profile of the cells. As mentioned previously, the miR-302 family were all reduced substantially to undetectable levels, given their specificity for ESC. Other ESC specific miRNA include miR-372 and miR-367, and these were also reduced to undetectable levels^27^.

miR-204 expression is dramatically increased, which has been implicated as the master regulator of retinal development. Over one third of the ~800 genes that miR-204 targets are expressed in the retina, and its targets have been implicated in a variety of diseases including the RGC pathology, glaucoma^28,29^. miR-125b was also found to be differentially abundant and has been implicated in non-neuronal retinal cells such as retinal pigment epithelium (RPE) differentiated from human ESC^30^. Interestingly, their study suggested that miR-125b and let-7 drove differentiation down a non-neuronal retinal lineage. Let-7a was another that was over-abundant in our ESC-RGC, and therefore our study demonstrates that these two (miR-125b and let-7a) are not RPE specific, but are instead also found in RGC. miR-125b is further upregulated in retinal cell death^31^. Finally, miR-125b has been implicated as a biomarker for glaucoma due to its differential expression in the aqueous humor of glaucoma patients compared to controls^32^.

The increase in expression of miR-181a corroborates literature on this miRNA being strongly expressed in the retina. Based on hybridization studies, the highest expression was in amacrine cells, but was also detectable in the ganglion cell layer^33,34^. Equally, in fish, miR-181a is required for the growth of amacrine processes and RGC axons^35^.

miR-125a was also overexpressed, but the literature has few details of its role in the eye, with some suggestion it is involved in RPE loss in cell lines after exposure to exosomes from dry AMD patients^36^ as well as retinoblastoma in mice^37^. miR-216b was overexpressed, and has been seen in the developing retina, driving differentiation of retinal neurons (and away from the retinal glia lineage) through a reduction in notch and Foxn3 signalling^38,39^. Finally, it’s been suggested to play a protective role in preventing diabetic retinopathy^40^, similar to miR-15b, another overexpressed miRNA^41^.

Recently, we published a miRNA sequencing analysis of purified RGC from rodents^12^. While many studies have sequenced the total retinal cell population, this is to our knowledge the only study that purified RGC. As such, comparisons with this study provide a unique assessment of the congruence in miRNA expression between rodent and human (ESC-derived) RGC. Of the 13 most abundant miRNA identified in our human ESC-RGC, 7 of these were also the most abundant miRNA identified in rat purified RGC, whereas 6 were only found in the human ESC-RGC (Table. 1). It is however unknown whether those miRNA unique to the human RGC are a consequence of species differences, or the cell culture environment in which the ESC-derived RGC were generated. Interestingly, miR-191 was flagged as a miRNA unique to (rat) RGC, not identified in other miRNA assays of other retinal cell populations^12^, and is also found to be abundant in the present study.

**Table 1:** The thirteen must abundant miRNA identified in human retinal ganglion cells (RGC), differentiated from embryonic stem cell H7/H9 line. Abundant is defined as a mean estimated abundance of over 2000. The miRNA are separated into those that were also identified in purified rat RGC, or those that were not, based on findings previously published (Mead et al*.*^12^).

The thirteen most abundant miRNA identified in human ESC-derived RGC	
miRNA also identified in rodent RGC	miRNA not identified in rodent RGC
miR-204-5p	miR-130a-3p
miR-1256-5p	miR-135a-5p
miR-181a-5p	miR-4454/7975
miR125a-5p	miR2166-5p
miR-25-5p	miR-93-5p
miR-191-5p	miR-15b-5p
Let-7a-5p	

Following differentiation and purification, it is unclear if the cells are stable or still undergoing changes. Comparing miRNA expression between 6 and 48 h in culture identifies several changes. While most of these changes are in low abundant miRNA, miR-135, -23a, and -107 all increased dramatically, whereas let-7b, -7d, -7i, -7 g, and -7 l (but not let-7a) all decreased dramatically. Let-7 is involved in neural progenitor development^42^, as well as late differentiation of both retinal neurons and glia^43^. It is thus tempting to speculate that the downregulation of let-7 is a consequence of maturation of the RGC. This is further corroborated by miR-135 being detectable in whole huma retina^44,45^. There are many subtypes of RGC within both animal and human retina, subtypes which so far have been relatively explored within rodent and human retinal organoids^46–48^. It would be of great use to further explore the subtype profile of these RGC, to determine if they belong to a distinct class of RGC or make up several expected subtypes.

In conclusion, the present study identifies the miRNA profile of both ESC and ESC-derived RGC, characterizing the differences. ESC-derived RGCs appear to express a variety of miRNA identified to be important in retinal development and function, while downregulating ESC-specific miRNA. Several of these miRNA have also been identified in purified rat RGC, however, six miRNA appeared to be unique to the human RGC. Further understanding of the biology of human RGC can be achieved by taking into consideration their unique miRNA signature.

## Supplementary Information

Supplementary Information.

## Data Availability

Data is provided within the manuscript, additional raw IPA data is available on request from corresponding author.
